# Supplementary material for: Asynchronous magnetic resonance elastography: Shear wave speed reconstruction using noise correlation of incoherent waves
Source: Magn Reson Med. 2022 Oct 27;89(3):990–1001. doi: 10.1002/mrm.29502 (PMC9792433; doi:10.1002/mrm.29502)
Supplement: Supplementary file 1 — DATA S1 MATLAB code used to generate the numerical simulations of Figure 1 [file MRM-89-990-s002.zip › k-Wave/helpfiles/example_ewp_layered_medium.html]

Explosive Source In A Layered Medium Example (k-Wave)


# Explosive Source In A Layered Medium Example

This example provides a simple demonstration of using k-Wave for the simulation and detection of compressional and shear waves in elastic and viscoelastic media within a two-dimensional heterogeneous medium. It builds on the Homogenous Propagation Medium and Heterogeneous Propagation Medium examples.

- Open the file in the MATLAB Editor
- Run the file in MATLAB

## Contents

- Defining the medium and source properties
- Running the simulation

## Defining the medium and source properties

In addition to the simulation functions for modelling compressional waves in fluid media (`kspaceFirstOrder1D`, `kspaceFirstOrder2D`, `kspaceFirstOrderAS`, and `kspaceFirstOrder3D`), k-Wave also includes functions for simulating the propagation of compressional and shear waves in isotropic elastic and viscoelastic media. These functions are based on the pseudospectral time domain (PSTD) method, and are called `pstdElastic2D`, `pstdElastic3D`. These are used in a very similar fashion to the fluid codes, and require four input structures which define the properties of the computational grid, the material properties of the medium, the properties and locations of any acoustic sources, and the properties and locations of the sensor points used to record the evolution of the wavefield over time. The `kgrid` and `sensor` inputs are defined in an identical fashion to the fluid code, while the `medium` and `source` input structures have slightly different field names to reflect the nature of solid materials.

In an isotropic elastic medium, the material properties can be characterised by the shear and compressional sound speeds, and the mass density. These are assigned to the `medium` structure as the fields `sound_speed_compression`, `sound_speed_shear`, and `density`. In a homogeneous medium, the material parameters are set as scalar values in SI units. In a heterogeneous medium, these are instead given as matrices with the same dimensions as the computational grid. In this example, a heterogeneous medium is created with two material layers.

```
% define the properties of the upper layer of the propagation medium
medium.sound_speed_compression = 1500 * ones(Nx, Ny);   % [m/s]
medium.sound_speed_shear       = zeros(Nx, Ny);         % [m/s]
medium.density                 = 1000 * ones(Nx, Ny);   % [kg/m^3]

% define the properties of the lower layer of the propagation medium
medium.sound_speed_compression(Nx/2:end, :) = 2000;     % [m/s]
medium.sound_speed_shear(Nx/2:end, :)       = 800;      % [m/s]
medium.density(Nx/2:end, :)                 = 1200;     % [kg/m^3]
```

For viscoelastic media, the absorption coefficient in units of dB/(MHz^2 cm) can also be defined for both compressional and shear waves. The elastic codes are based on the classical Kelvin-Voigt absorption model, which gives absorption proportional to frequency squared in the low frequency limit.

```
% define the absorption properties
medium.alpha_coeff_compression = 0.1;   % [dB/(MHz^2 cm)]
medium.alpha_coeff_shear       = 0.5;   % [dB/(MHz^2 cm)]
```

The elastic codes support three types of sources: (1) an initial pressure distribution, (2) time varying velocity sources, and (3) time varying stress sources. These are used in an analogous fashion to the fluid codes. In this example, an explosive source is assigned to `source.p0`. Within the simulation function, this is then assigned to the normal components of the stress.

```
% create initial pressure distribution using makeDisc
disc_magnitude = 5; % [Pa]
disc_x_pos = 30;    % [grid points]
disc_y_pos = 64;    % [grid points]
disc_radius = 5;    % [grid points]
source.p0 = disc_magnitude * makeDisc(Nx, Ny, disc_x_pos, disc_y_pos, disc_radius);
```

## Running the simulation

The elastic codes are based on the pseudospectral time domain method. This does not use a k-space corrected scheme for time integration like the fluid codes, which means smaller time steps are typically required for stable and accurate simulations. In this example, the Courant-Friedrichs-Lewy (CFL) number is set to 0.1.

```
% create the time array
cfl   = 0.1;    % Courant-Friedrichs-Lewy number
t_end = 8e-6;   % [s]
kgrid.makeTime(max(medium.sound_speed_compression(:)), cfl, t_end);
```

The simulation is then started by calling `pstdElastic2D` with the four input structures analogous to `kspaceFirstOrder2D`. Optional inputs can similarly be defined as `'string', value` pairs after the main inputs. By default, as the simulation runs, a visualisation of the propagating wave field and a status bar are displayed with frame updates every ten time steps. Both the normal and shear components of the stress are displayed. The plot scales for these can be defined individually using the `'PlotScale'` input. This is defined as `[sii_min, sii_max, sij_min, sij_max]`, where `sii` and `sij` denote the plot scales for the normal and shear stress.

```
% define input arguments
input_args = {'PlotScale', [-0.75, 0.75, -0.15, 0.15], 'PlotPML', false,...
    'DisplayMask', display_mask, 'DataCast', 'single'};

% run the simulation
sensor_data = pstdElastic2D(kgrid, medium, source, sensor, input_args{:});
```

A visualisation of the running simulation and the recorded sensor data are given below. Both compressional and shear waves in the lower layer of the medium are clearly visible.
